# Red color facilitates the detection of facial anger — But how much?

**DOI:** 10.1371/journal.pone.0215610

**Published:** 2019-04-17

**Authors:** Tarja Peromaa, Maria Olkkonen

**Affiliations:** 1 Department of Psychology & Logopedics, University of Helsinki, Helsinki, Finland; 2 Department of Psychology, Durham University, Durham, United Kingdom; University of Sussex, UNITED KINGDOM

## Abstract

The color red seems to be consistently associated with the concept of anger. Beyond semantic associations, it has been suggested that the color red enhances our ability to perceive anger in faces. However, previous studies often lack proper color control or the results are confounded by the presence of several emotions. Moreover, the magnitude of the (potential) effect of red has not been quantified. To address these caveats, we quantified the effect of facial color and background color on anger with psychometric functions measured with the method-of-constant-stimuli while facial hue or surround hue was varied in CIELAB color space. Stimulus sequences were generated by morphing between neutral and angry faces. For the facial color, the average chromaticity of the faces was shifted by ΔE 12/20 in red, yellow, green and blue directions. For the background color, the hue was either neutral or saturated red, green or blue. Both facial redness and surround redness enhanced perceived anger slightly, by 3–4 morph-%. Other colors did not affect perceived anger. As the magnitude of the enhancement is generally small and the effect is robust only in a small subset of the participants, we question the practical significance of red in anger recognition.

## Introduction

We intuitively associate red with danger, anger, and arousal [1]. These associations seem to be consistent across cultures [2] as well as across species, as illustrated by the warning and socio-sexual displays of many animals (e.g. [3, 4, 5, 6]). Even the evolution of language points to a role of red as an important signal color: red is consistently the first chromatic color category to emerge after a light/dark or warm/cool split [7, 8, 9].

Common metaphors hint at a particular relationship between red and the feeling of anger: angry people see red or shout red-headed, depending on the language of the speaker. Several studies have shown a conceptual association between red and anger [2, 10, 11, 12, 13], but there is also evidence for a more specific effect of red on emotion recognition (e.g. [14, 15, 16, 17, 18]). For instance, facial redness increases the proportion of angry responses to neutral faces [14] and the rated anger of synthetic male faces [15]. Facial redness might also affect category boundaries between anger and other emotions, such as anger and fear ([17, 19]; although see [14]).

Is there a physiological basis for the connection between red and anger? Darwin [20] noted that the face reddens or becomes purple during rage, with distension of forehead and neck veins–in other words, the face flushes. Flushing is due to a rush of oxygenated blood to the facial veins, which is perceived by trichromatic humans as increased redness [21, 22, 23]. Given that there is a physiological link between facial redness and anger, using facial color to interpret the emotional state of conspecifics appears a useful strategy to reduce uncertainty in the emotion estimate. But more counter-intuitively, even a red background appears to have an effect on emotion recognition; classification of anger from faces is facilitated by a red background in terms of decreased reaction times [24] or by increasing “angry” responses to neutral faces [25]. Furthermore, Wiedemann, Burt, Hill and Barton [26] found that red clothing increased the rated aggression, dominance, and anger of male faces. Whether the effects of facial color and contextual color on perceived emotion are actually caused by the same underlying mechanism is nevertheless unclear.

Although the literature to date points to a relationship between the color red and perceived emotion, several caveats complicate the interpretation of the results as a whole. First, comparing stimulus conditions across studies is difficult because most studies do not report performing color calibration. Second, several studies employ only two colors [24, 25, 27], which might confound general saliency effects with the effects of red specifically, and might serve to reveal the hypothesis to the participants. Third, some studies are confined to neutral faces only [16, 25], and it is not clear whether the results would generalize to angry faces, where featural information about the emotion is available. And finally, the most commonly used methods do not allow for quantifying the magnitude of the effect. For example, knowing that participants classify neutral faces as “negative” more often than “positive” does not tell us how much angrier the face in fact appears in this particular condition. To be able to assess the practical significance and, eventually, the neural mechanisms of these color-mediated effects on emotion recognition, it is pivotal to address these pitfalls.

Here we employ standard psychophysical methods to test the hypothesis that color affects the perception of emotion. This allows us to quantify the magnitude of the effect under carefully controlled viewing conditions. More specifically, we measure psychometric functions to derive quantitative thresholds for perceiving anger under different face and background color conditions. We focus on the effect of red on the perception of anger because these have been most often paired in the literature, and because of the postulated evolutionary importance of the color red (see e.g. [23]). However, to mitigate general saliency effects and possible observer strategies we employ other cardinal colors also. To anticipate, we find a reliable but small effect of facial redness on the detection of anger and a trend of an effect for red background. However, we argue that the practical significance of this effect is limited because of its small magnitude.

## Methods

### Equipment

The stimuli were presented on a 23” VIEWPixx/3D TFT LCD–display with a LED backlight. The resolution of the display was 1920x1080 px (active area 51.7x29.1 cm) and the refresh rate 120 Hz. The display was color calibrated to maximum luminance of 250 cd/m^2^ and white point corresponding to D65. The viewing distance, controlled with a head rest, was 95 cm. The experiments were run in Matlab-environment (Version 8.5 R2015a, Mathworks, Inc.), using Psychtoolbox-extensions (Version 3.0.12) [28, 29, 30].

### Participants

There were 24 participants (age 18–50, median 22 years, 18 female and 6 male); 4 were removed from the analysis because of poor discrimination of expression. (Some participants were unable to perceive anger in one of the stimulus identities; the problematic identity varied across participants. This showed as a truncation of the psychometric functions, i.e. the functions saturated around 75% probability of responding “angry” instead of reaching 100%. Such functions do not serve well for estimating thresholds.) Written informed consent was obtained from all participants prior to testing. The participants had normal or corrected-to-normal vision and no reported problems with color vision. They were naive with respect to the purpose of the experiment. All the participants were Caucasian. The study was carried out in accordance with Declaration of Helsinki, and approved by the University of Helsinki Ethical Review Board in the Humanities and Social and Behavioural Sciences.

The number of participants was chosen on the basis of a power analysis applied to pilot data with the aim of 0.8 power at a medium effect size.

### Stimuli

Eight Caucasian facial stimuli were chosen from The Umeå University Database of Facial Expressions [31]: neutral and angry expression from 4 individuals (2 male). The ear-to-ear width of the faces was equalized to 420 pixels. One of the individuals had hair falling onto the forehead, which did not allow for neat morphing; thus, for this individual the hairline from the neutral expression was copied onto the angry expression.

A binary mask was generated for each face for the analysis and adjustment of facial color. The analysis mask contained facial skin area, excluding eyes, mouth, eyebrows, nostrils and hair. The adjustment mask contained facial skin area, excluding eyes and hair. The masks were generated with Adobe Photoshop CS6.

The color manipulations were accomplished in Matlab, with custom-built routines.

The images were converted to CIELAB from sRGB, assuming illuminant d50 (the details of the original lighting are not given in the database—this was the best guess) and with gamma correction (gamma 2.4). Face color was extracted from the CIELAB image using the analysis mask. The face color was then adjusted (using the adjustment mask) to the chromaticity of average Caucasian skin a*/b* 11.7/ 14.85, taken from the spectrophotometric database for human skin by Xiao & al [32]. The average L*-value of the faces in the sample was 69.4. This is much higher than the average Caucasian L*-value in the Xiao & al [32] sample, which was 59.4. On a calibrated display, the faces appeared too light. Because of this, the L*-value was reduced by 10 units across the whole stimulus (including the eyes, hair & background).

**Fig 1:**
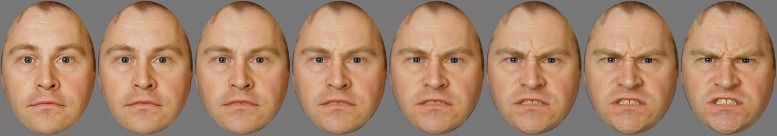
Morph sequence from neutral to angry expression (0–100%). The identity shown is a demo identity, not used in the actual experiments. All the identities used in the experiments had a closed-mouth expression of anger; however, the use of those images in publication is not permitted. The image of the demo identity is derived from [31] under a CC BY license, with permission from P Carlbring, original copyright 2012 (Hanna Samuelsson, Karl Jarnvik, Hanna Henningsson, Josefin Andersson, Per Carlbring).

A morph sequence between the neutral and angry expression (Fig 1) was generated for each of the four individuals with FantaMorph Deluxe 5 (Abrosoft, Nebraska, US). The morphing was done in the L*a*b* domain. The stimuli were windowed after morphing (431x606 px ≈ 7.0x9.7 deg).

### Experiment I: The effect of facial color

**Fig 2:**
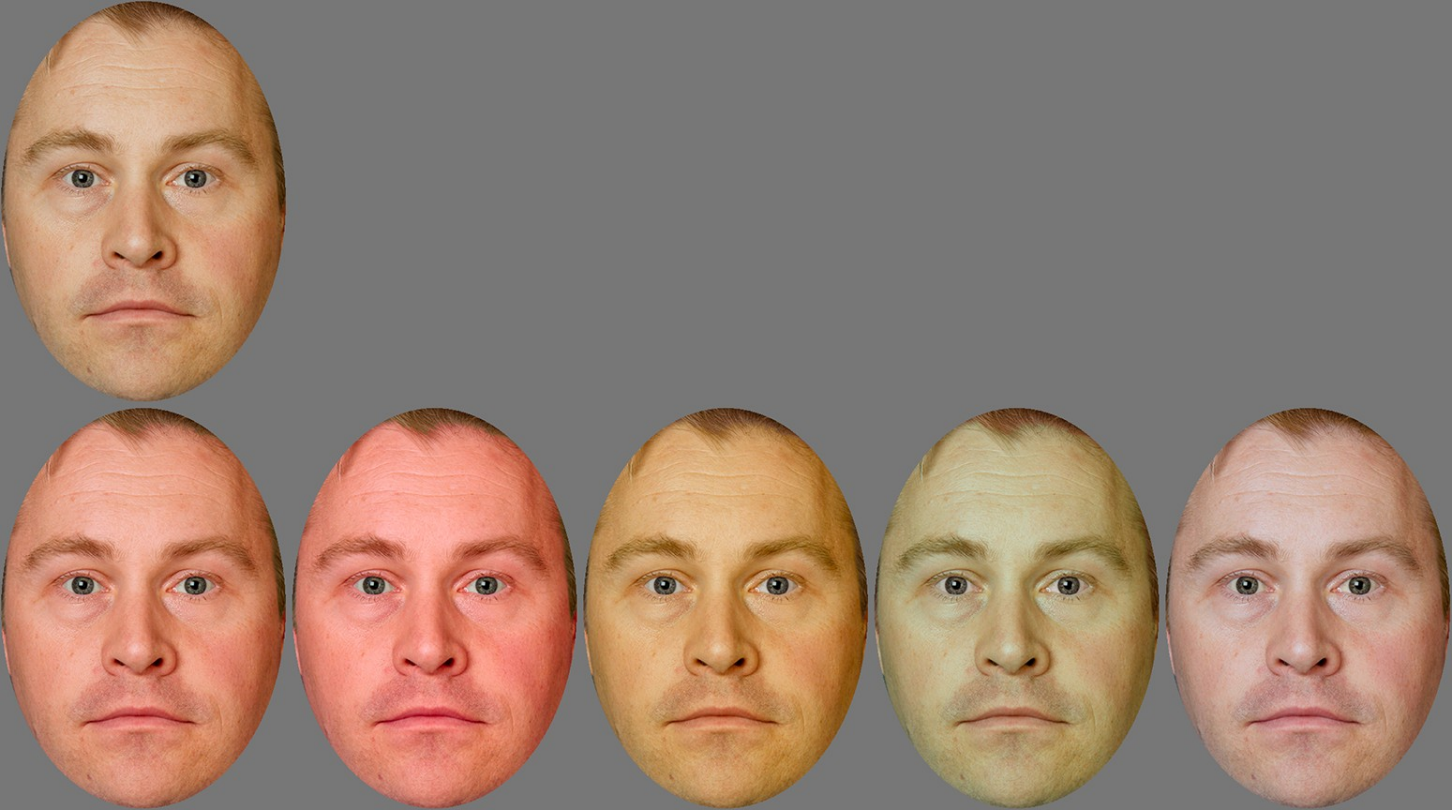
The facial color conditions. Top: baseline; bottom: red, red+, yellow, green, blue. Note that colors in this and following figures are only approximate on non-calibrated display media.

Five different color conditions were created by adjusting the facial color (a*b*) from the original (baseline) (Fig 2). Four conditions were created by shifting the face color by ±12 units in four directions in the a*b* plane (red, yellow, green, blue); for the fifth condition (red+), ΔE 20 was applied in the 0° (“red”) direction (see Supplementary Table A in S1 Text online). The five conditions were chosen on the basis of a pilot experiment, where the a*b*-space was sampled with a 45° resolution of the hue angle. The directions chosen (0°, 90°, 180° and 270°) correspond to the CIELAB cardinal axes, as the intermediate hue angles did not produce changes to the thresholds. We added the red+ condition to maximize our ability to detect an effect of red on anger detection. The stimuli were presented on a neutral gray background (L* = 50; a* = b* = 0).

### Experiment II: The effect of background color

**Fig 3:**
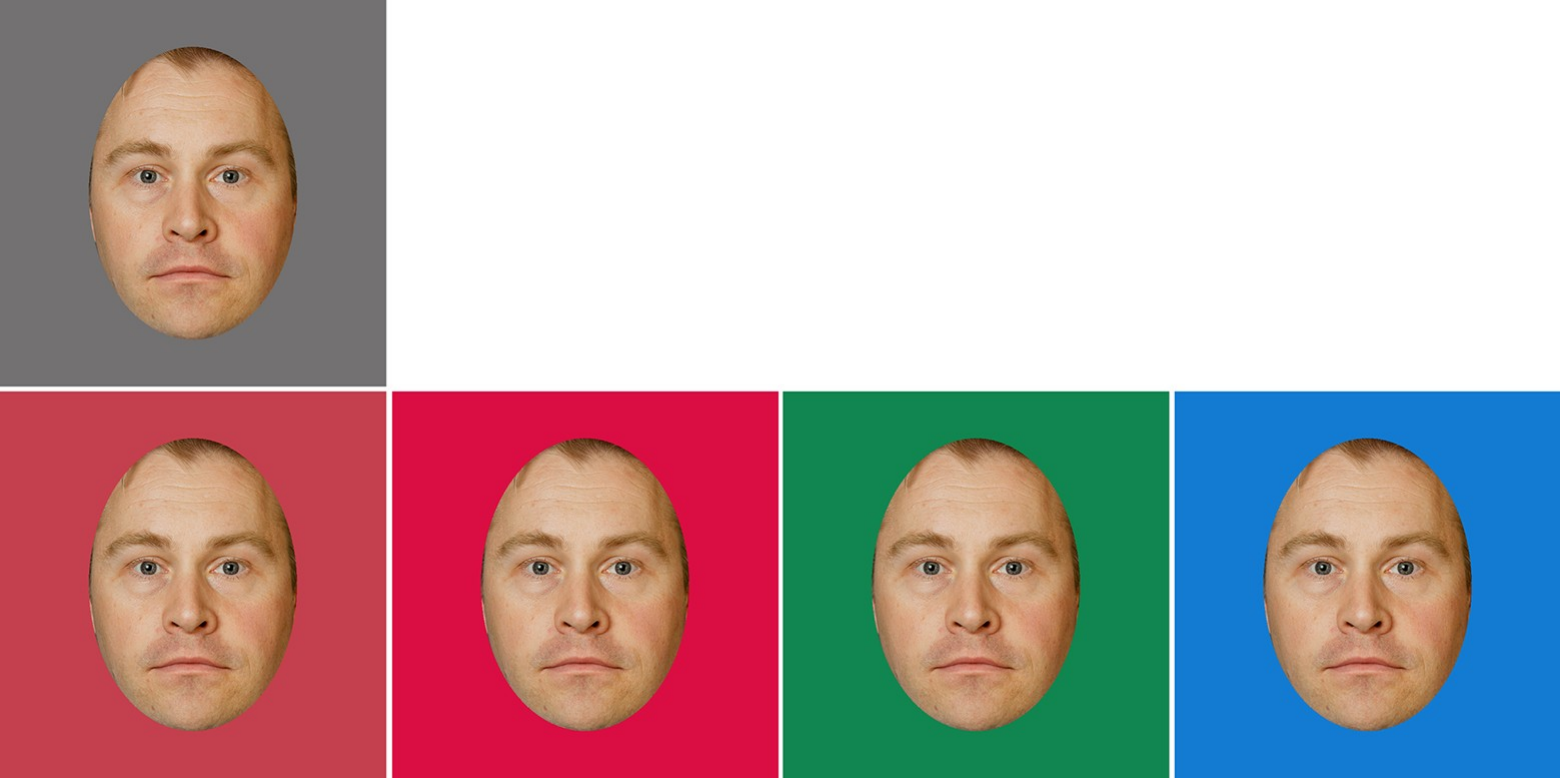
The background conditions. The neutral baseline condition on the top and the red, red+, green and blue conditions below.

Five different background color conditions, in addition to the achromatic control condition (baseline), were employed (see details in the Supplementary Table B in S1 Text online). The background colors were chosen from the hue angles corresponding to unique hues: red, green and blue (Fig 3). (In a pilot experiment, also hue angles corresponding to the red-green and blue-yellow axes of the DKL color space were studied, but those did not induce any changes.) Lightness was set to L* = 48 and saturation to S = 60. For the red background color, there was an additional condition (red+) with saturation set to a higher value, S = 80 (see Supplementary Table B in S1 Text online), assuming that we should see a stronger effect with more saturated red. There was no yellow background, as it would have required a higher L*-level than the one employed in this experiment.

The size of the background was 800x800 px (12.9x12.9 deg). The display background was achromatic (L* = 50; a* = b* = 0).

### Procedure

The psychometric functions for the recognition of anger were measured with the method-of-constant-stimuli for each face color and each background color in separate blocks.

On a single trial, a facial stimulus was presented for 500 ms. The task of the participant was to indicate, by pressing the appropriate button, whether the face was angry or not. The location of the stimulus on the display was slightly jittered (± 25 px) trialwise to minimize direct comparison of local features when judging the emotion. The next trial started 1000 ms after the participant had responded. The response time was also recorded.

Each psychometric function was measured with eight morph levels and four identies. Each morph level and identity were presented five times, giving 20 repetitions per morph level (across identity). Both experiments were divided into five blocks, each containing either 192 (facial color: 6 conditions x 8 levels x 4 replications) or 160 (background color: 5 conditions x 8 levels x 4 replications) trials, in random order. The five face color and five background color blocks were run interleaved in one session, which took 1–1 ½ hours.

### Analysis

The relative number of yes-responses was calculated for each morph level, for each condition. A cumulative normal distribution (CND) was fitted to the proportion “angry” responses using the Palamedes toolbox [33] with the Maximum Likelihood criterion. The mean (50% threshold) and 1 / standard deviation (slope) of the CNDs were analyzed.

The data were tested for normality using Shapiro-Wilk test. Assumption of normality satisfied, repeated measures ANOVA with planned contrasts was used to test whether facial or background color induced a change in emotion identification from the neutral baseline condition. When not, a non-parametric Friedman’s test was used instead. Multiple comparisons for the planned contrasts were corrected for by dividing the *α* (0.05) with the number of the comparisons (Bonferroni method). Additionally, Bayesian t-tests were applied to gain insight into non-significant contrasts [34]. Bootstrapping [35] was used to derive the 95% confidence intervals for the color-induced changes participantwise. The data for each participant’s psychometric functions was resampled (with replacement) 1000 times, keeping the total number of trials the same as in the experiment.

## Results

### Experiment I: Face color

**Fig 4:**
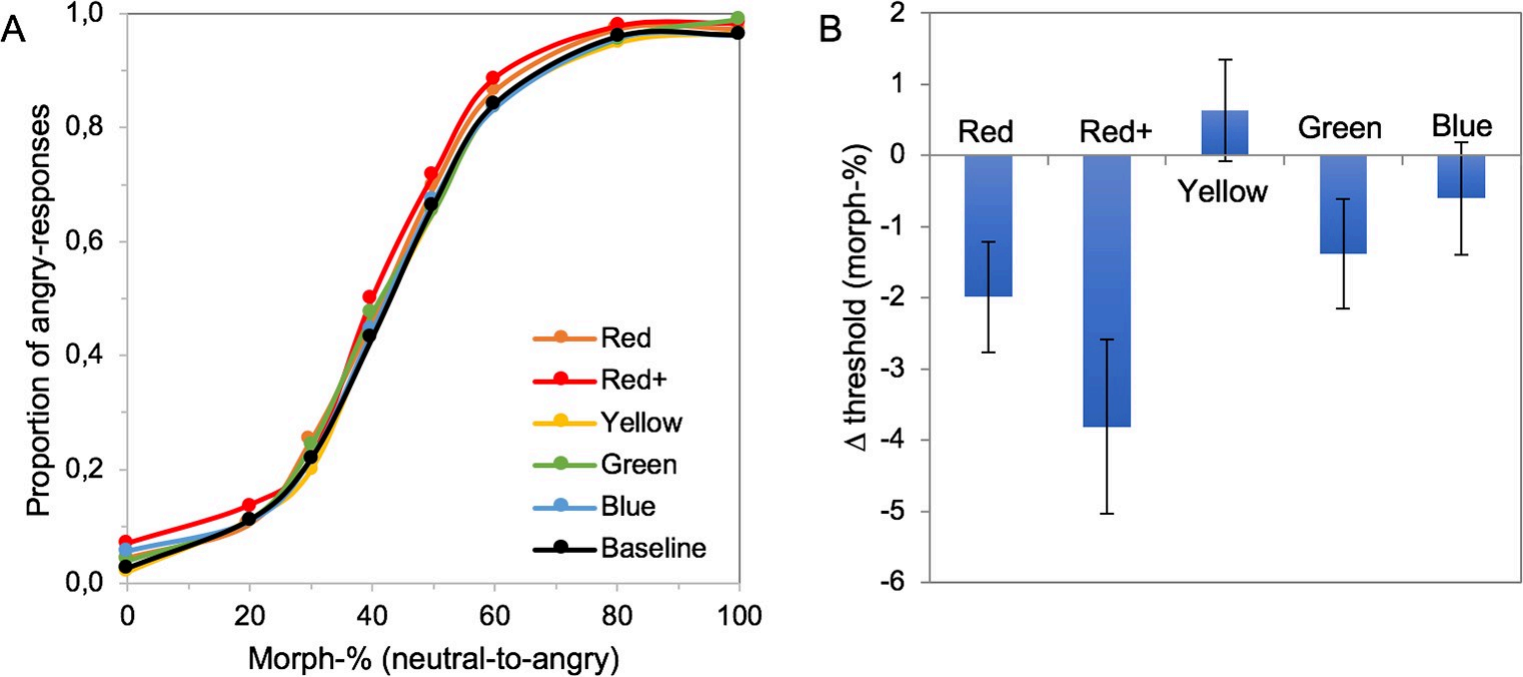
The effect of facial color on anger perception. (A) Psychometric functions showing anger recognition performance as a function of morph level for each facial color condition, averaged across participants (N = 20). (B) Average color-induced change in the 50% threshold (i.e. chromatic—baseline threshold) for each facial color. Error bars represent +/- 1 standard error of the mean across 20 participants.

The psychometric functions for the different facial color conditions, averaged across participants, do not differ much, as is evident from Fig 4A. However, the changes induced by the color are here masked by the large individual differences in the sensitivity to the emotion. For example, under the neutral baseline condition, the 50% threshold to detect anger varied between 20 and 62 morph-%. Quantifying performance in the chromatic conditions as a function of baseline performance for each participant shows that facial color affected the thresholds for the detection of anger (Fig 4B). A 1-way repeated measures ANOVA with different face colors as factor revealed a main effect of facial color on anger detection (F(5,95) = 4.984, p < .001, $\eta_{partial}^{2}$ = .208). Planned contrasts (baseline vs the 5 color conditions) showed that the decrease in threshold relative to baseline for red+ (F(1,19) = 9.644, p = .006, $\eta_{partial}^{2}$ = .337) was significant. The decrease was on the order of 4 morph-%. For the standard red, the decrease did not remain significant after the correction for multiple comparisons (F(1,19) = 6.515, p = .019; *α* 0.01). The difference between standard and saturated red was not significant (t(19) = 2.025, p = .057 (2-sided), achieved power = .485)–but possibly suggests a trend for increasing effect with increasing saturation. A Bayesian t-test exploring this difference yielded an inconclusive Bayes factor (JZS Bayes factor B_01_ 1.023), not providing strong evidence for or against a difference. Other colors did not induce significant changes in the threshold (t-values < 1.796 and p-values > .088). In summary, participants were significantly more likely to indicate the presence of anger for reddish faces, but the effect was relatively small.

The slopes of the PMFs were not affected by facial color (χ^2^(5) = 3.312, p = .652), suggesting that subjects were equally able to discriminate the intensity of the emotion in all conditions.

Average response time in the experiment was 759 ms; facial color did not significantly affect the response times (F(2.973,56.492) = 2.403, p = .078).

### Experiment II: Background color

**Fig 5:**
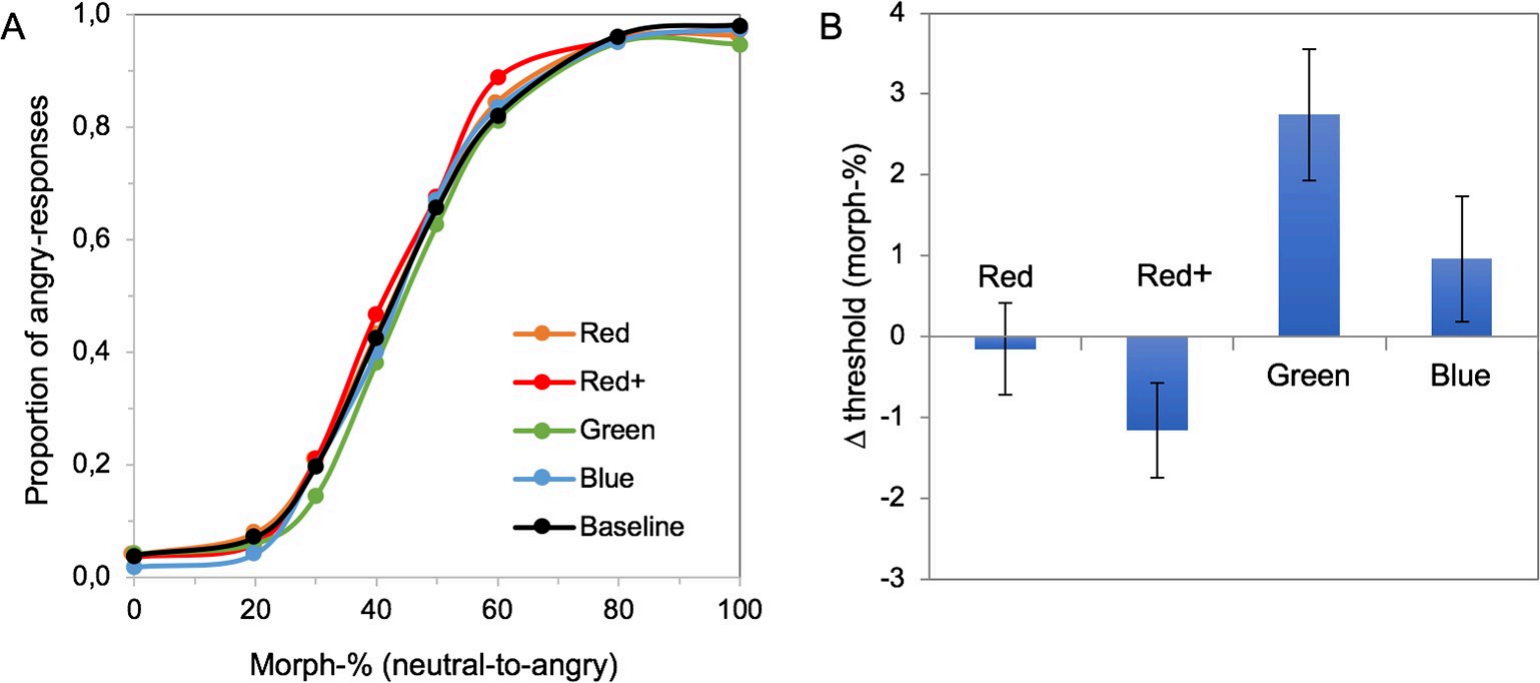
The effect of background color on anger perception. (A) Psychometric functions showing anger recognition performance as a function of morph level for each background condition, averaged across participants (N = 19). (B) Average color-induced change in the 50% threshold for each background color. Error bars represent +/-1 standard error of the mean across 19 participants.

Background color affected the thresholds for the detection of anger (Fig 5). A 1-way repeated measures ANOVA with background color as factor revealed a main effect for background color (F(4,72) = 7.085, p < .001, $\eta_{partial}^{2}$ = .288). Based on preliminary checks, one participant was removed from the analysis as an outlier (the detection threshold in the baseline condition 3 SDs below the average), causing non-normality of the data; the removal did not affect the general pattern of results. Planned contrasts (baseline vs the 4 color conditions) showed that the effect of green was significant: F(1,18) = 11.481, p = .003, $\eta_{partial}^{2}$ = .389. The thresholds increased by ≈3 morph-%, showing that the participants were less likely to indicate the presence of anger when the background was green. Other colors did not induce significant changes in the anger detection threshold (t-values < 2.001 and p-values > .061).

The slopes of the PMFs were affected by the background color (χ^2^(4) = 10.160, p = .038). However, the pairwise comparisons did not reveal any significant differences between the baseline and the chromatic conditions.

Average response time in the experiment was 771 ms; background color did not significantly affect the response times (F(4,76) = .265, p = .900).

Why was there an inhibitory effect for the green background, and no effect for red? Based on previous literature and our Experiment 1 we might have expected a facilitatory effect for red and no effect for the other colors. Upon closer scrutiny, the baseline condition used here may not have been adequately matched to the chromatic condition. This is because the overall color difference between the local background (immediately around the faces) and the larger, global background was much greater for the chromatic backgrounds than for the baseline background. In the baseline condition there was only slight luminance contrast (ΔL* = 2) at the local/global background edge, whereas in the chromatic conditions there was a large chromatic contrast component in addition to the weak luminance contrast component. Thus, the edge of the stimulus was much more salient under the chromatic conditions, orienting attentional resources more efficiently (e.g. [36]). Quite possibly, the more salient (spatially peripheral) edge in the chromatic conditions drew attentional resources from (spatially central) facial stimuli more than in the baseline condition, drowning out any facilitatory effects of a red background. To control for this, we repeated Experiment II with slightly modified parameters.

### Experiment IIb

Experiment IIb was otherwise identical to Experiment II, but now the global background luminance was decreased from L* 50 to 25. This manipulation provided a large edge transient also for the baseline condition, rendering the different conditions more comparable to each other.

A new group of subjects was recruited. There were 21 Caucasian participants (age 18–43, median 22.5 years, 19 female and 2 male); 1 was removed from the analysis because of poor discrimination of expression (see above for the exclusion criteria).

### Results

**Fig 6:**
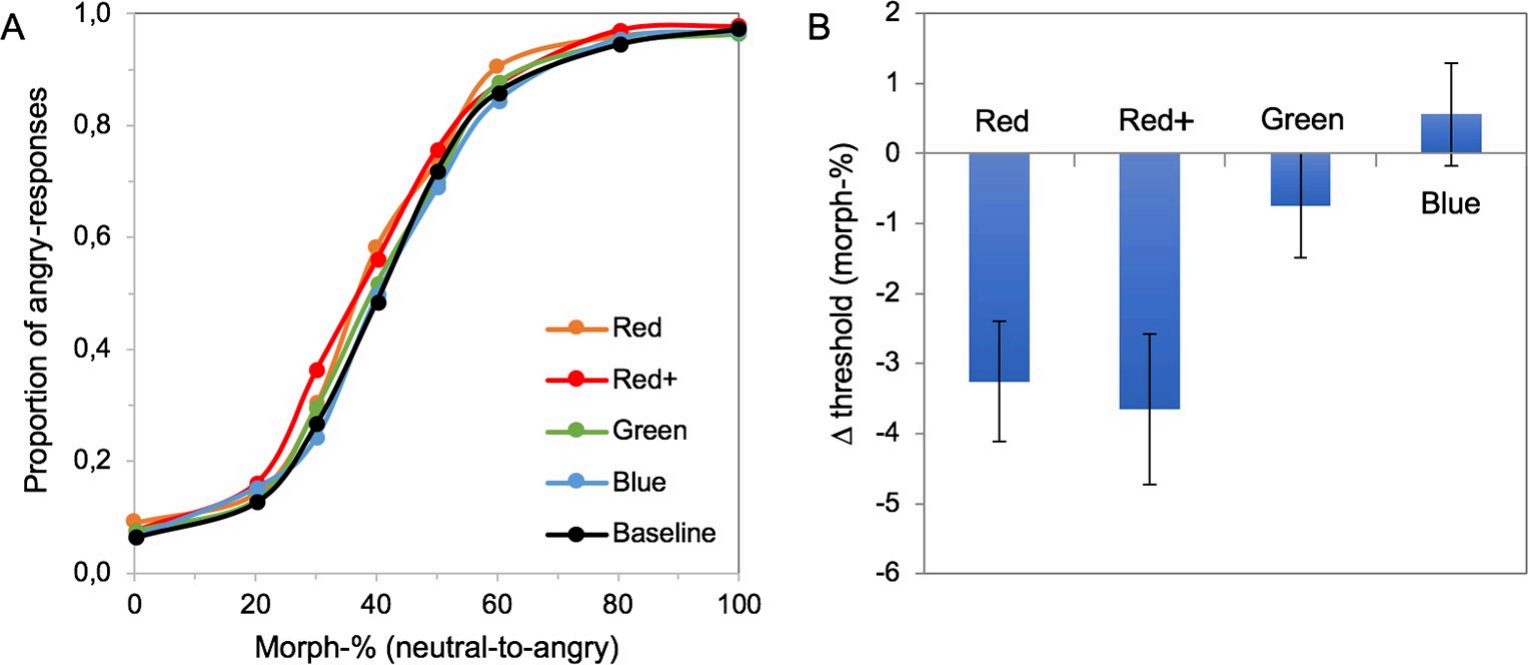
The effect of background color on anger perception. (A) Psychometric functions showing anger recognition performance as a function of morph level for each background condition, averaged across 20 participants. (B) Average color-induced change in the 50% threshold for each background color. Error bars represent +/- 1 standard error of the mean.

Again the background color affected the thresholds for the detection of anger (1-way repeated measures ANOVA, F(4,76) = 9.264, p < .001, $\eta_{partial}^{2}$ = .328), but the pattern of results was different from the first version of the experiment (Fig 6). Planned contrasts showed that both red backgrounds significantly facilitated anger detection: F(1,19) = 14.347, p = .001, $\eta_{partial}^{2}$ = .430 and F(1,19) = 11.479, p = .003, $\eta_{partial}^{2}$ = .377 for the standard and saturated red, respectively. The thresholds decreased by 3–4 morph-%, showing that the participants were more likely to indicate the presence of anger when the background was red. The difference between the two red conditions was not significant (t(19) = .509, p = .617, achieved power = .0772), and the Bayes factor was in favor of the null (JZS Bayes factor B_01_ 5.18). Other colors did not induce significant changes in the threshold (t-values < .989 and p-values > .335).

Again, the slopes of the PMFs were not affected by the background color (F(4,76) = .673, p = .613).

Average response time in the experiment was 679 ms; background color did not significantly affect the response times (F(4,76) = .388, p = .816).

To compare the effect magnitude for facial color compared to background color, we ran a 2-way mixed-effects ANOVA with saturation of the red color (red vs. red+) as a within-subjects factor and experiment type (face vs. background color) as a between-subjects factor. There was no difference in the magnitude of the effects in the two experiments (F(1,38) = .186, p = .668, observed power = .071). There was no main effect for the saturation of the red (F(1,38) = 3.468, p = .070, observed power = .442), nor interaction between the experiment type and the saturation (F(1,38) = 1.433, p = .239, observed power = .215); note however that the observed power of the tests was low.

**Fig 7:**
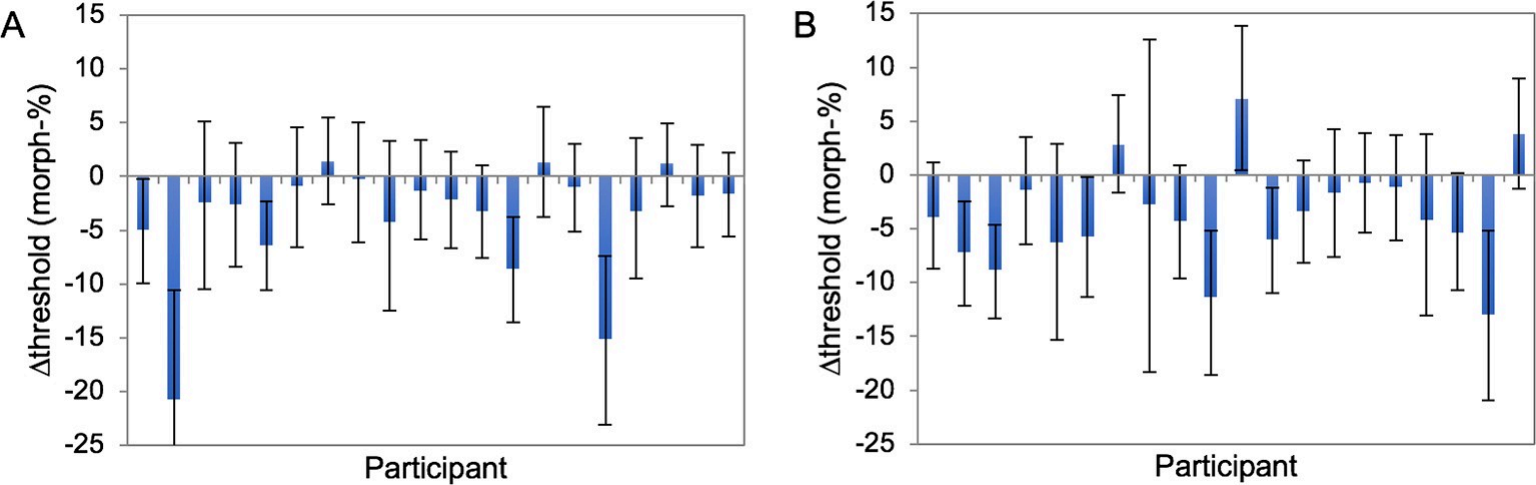
The threshold change induced by the more saturated red color, for all participants. (A) Facial color condition, (B) background color condition (exp IIb). Error bars represent the 95% confidence intervals derived from bootstrapping.

Although we did find the enhancing effect of the color red on the perception of anger at the group level, the magnitude of the effect was not very large; only 3–4 morph-%. Fig 7 shows the change in threshold for the saturated red relative to baseline with bootstrapped 95% confidence intervals for each participant. It is evident that the confidence intervals do not include zero in only 5–6 participants in each experiment, suggesting that the enhancing effect is robust only for a small subset of participants. It is worth noting however that no participant showed the opposite effect reliably either.

## Discussion

We studied the effect of facial and background color on the perception of anger by characterizing anger detection thresholds in color-calibrated stimulus displays. Red facial and background color enhanced the perception of anger by 3–4 morph%, while yellowish, greenish or bluish facial or background color did not have an effect on thresholds. This effect was significant but relatively small and robust only for a minority of the participants. We discuss the results for facial and background color separately first, and then compare the two. Finally, we assess the practical significance of the effect.

We found red facial color to enhance the perception of anger by 3–4 morph-%. Previously, Young et al [15] showed that red synthetic faces are rated slightly (up to ≈ 7%) more angry than neutrally colored faces. They varied facial color only on the a*-axis (red) of CIELAB space, which may have given cues about the hypothesis for the participants. Young et al found that redness had no influence at low a* levels, but increased the rated anger linearly at higher a* levels (> 20). Thorstenson, Pazda, Young and Elliott [36] found a similar result: Synthetic faces morphed between an angry and disgusted expression were rated increasingly angry as a* was increased above the original value (which was not reported). We found the enhancing effect at a* = 23.7 and 31.7. We thus validate the results of Young et al [15] and Thorstenson et al [37] using natural stimuli and a wider palette of colors.

The effect we found was relatively small, and it is possible that we would have obtained a larger effect with more extreme a* values. We chose the most saturated a* value by eye so that it still appeared relatively natural to us; we deemed levels beyond 32 a* units visually too extreme. There is no objective way to determine acceptable a* levels; although anger-induced flushing and accompanying changes in blood flow have been measured [21, 22], as far as we know, the magnitude of the accompanying color change has not been characterized. Based on previous results and those of ours, we expect the red-induced effect to be well below 10 morph-% for any reasonable level of facial redness.

Previously, Minami and colleagues [17, 19] studied the effect of red on emotion perception in a setup similar to ours. They showed that red facial color shifts the category boundary on a fear-anger continuum away from anger, thus increasing the probability of reported anger. The shift is in the direction of facilitation of anger and in this sense, in line with our results; however, the magnitude and cause of the shift is difficult to interpret, as there are two emotions potentially affected by the color. The red color might enhance the perception of anger, or inhibit the perception of fear, or both. In other words, rather than showing directly how the color red affects the perception of anger, these results show effects on the perception of anger in relation to fear.

Surprisingly to us, we found an effect of a red background on the perception of anger on the order of 3–4 morph-%. This is unexpected if one considers basic perceptual mechanisms, as it is against a simple prediction from simultaneous color contrast (see e.g. [38]). According to this well-known color context effect, the red background should induce a greenish tinge into the neutral face and thus decrease the perceived redness of the face, presumably decreasing, rather than enhancing, perceived anger. Our result goes against this prediction and complements previous reports of a facilitatory effect of a red background on reaction times [24], biases for ambiguous expressions [25], or shifts in a category boundary between two emotions [19].

Are the effects of facial and background color related? We did not find any significant difference between the magnitudes of the two effects, although there was a tendency for the background color effect to be slightly larger. Minami et al [19] found the opposite: facial color exerted a larger effect on emotion perception than background color. It appears, on the basis of previous studies [15] and the trend in our data, that the magnitude of the effects may depend on the stimulus parameters; particularly, on the saturation of the colors. For facial redness, we found a possible trend pointing towards increasing effect with increasing saturation. Interestingly, for the background redness, the effects were unaffected by the saturation. A possible scenario is that the effects of background color are categorical, whereby the activation of “anger” by “red” at the conceptual level might lead to a general criterion shift, regardless of the strength of the color cue. The effects of facial color might be more continuous, reflecting rather a perceptual integration of the color cue with the featural cue. In this scenario, the strength of the color cue would affect its weight in the integration process, which would produce a monotonously increasing relationship between color saturation and sensitivity to anger from faces. The results so far do not allow us to draw firm conclusions about the underlying mechanisms of these two effects, and this remains an intriguing topic for future research.

**Fig 8:**
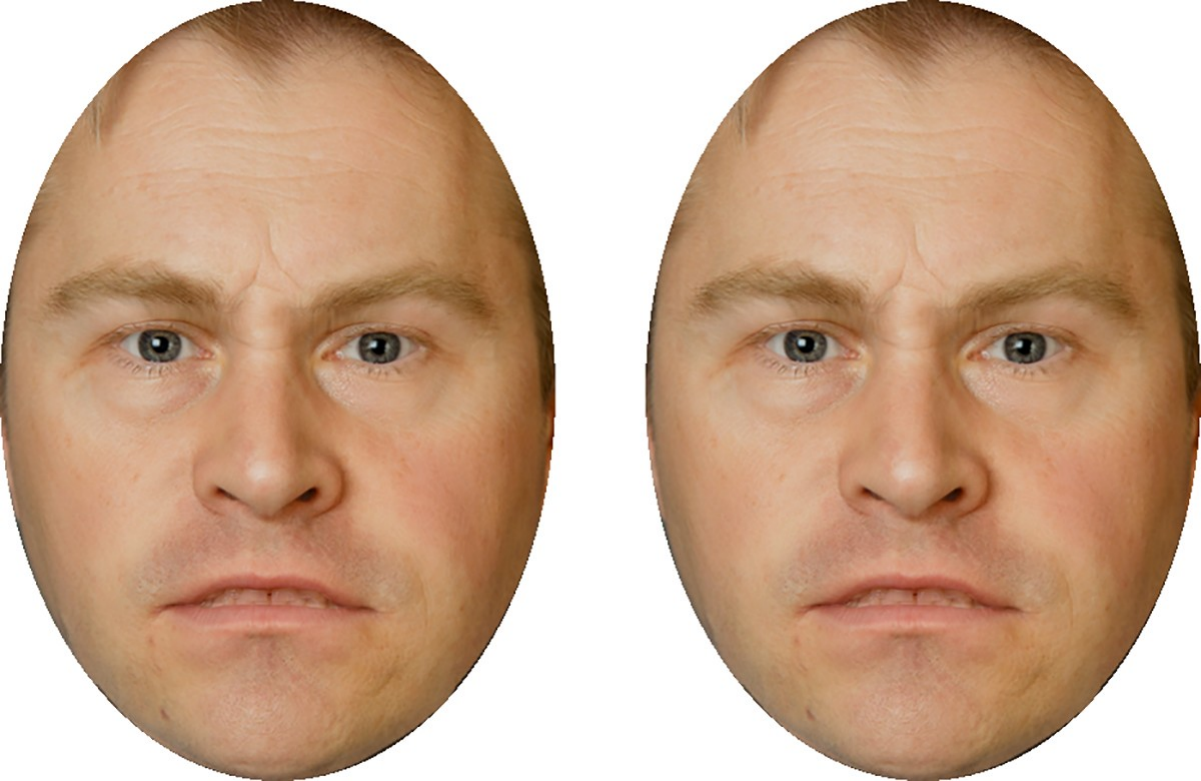
Demonstration of the magnitude of the modulation. 40 vs 45%, i.e. 5 morph-% difference in the intensity of emotional expression.

Are the reported effects significant for practical purposes? The effect sizes as quantified by partial *η*^2^ were large (all $\eta_{partial}^{2}$ > 0.14), but the magnitudes of the measured effects in the stimulus space were less than 5 morph-%. For a visual demonstration of the effect, Fig 8 shows two faces with a 5 morph-% difference in the intensity of the angry expression. Casual inspection of these images reveals that the effect is there, but it seems slight. The effect magnitude also seems modest in comparison to emotion adaptation effects, which have been found on the order of 10–20 morph-% (e.g. [39, 40]). All of this implies that these effects are likely to be of limited practical importance in most situations.

Two recent studies have speculated about the role of facial color in expression perception [37, 41]. These studies claim that 1) facial color facilitates the disambiguation of confusing emotion expressions [37] and that 2) facial color per se is an efficient mechanism to visually transmit emotion, even without any featural cues [41]. The slight modulation of emotion perception we found seems incompatible with the idea that color independently and efficiently provides emotional information. Our results are more compatible with the idea that color serves a facilitatory (or assisting) role, being one cue among many for emotion identification. Using color together with featural information when identifying facial expressions would be a good strategy to reduce uncertainty in the perceptual estimate, especially if featural information is noisy or variable. In most cases, however, featural information is a more salient and reliable cue than color, so it should be weighted more, in line with Bayesian models of cue integration (e.g. [42]).

In summary, we find that red color on a face and in the background make faces appear angrier. While the effect is statistically robust, it is visually small. The effect is likely to be of small practical significance.

## Supporting information

S1 Text Table A. The average facial colors in the six conditions. Table B. Hue angles and a*b*-coordinates of the background color conditions.
(DOCX)

S1 Dataset The means and slopes of the psychometric functions and the average response times for the individual participants. (XLSX)
