# Supplementary material for: Red color facilitates the detection of facial anger — But how much?
Source: PLoS One. 2019 Apr 17;14(4):e0215610. doi: 10.1371/journal.pone.0215610 (PMC6469786; doi:10.1371/journal.pone.0215610)
Supplement: S1 Text — Table A. The average facial colors in the six conditions. Table B. Hue angles and a*b*-coordinates of the background color conditions. (DOCX) [file pone.0215610.s001.docx]

**Supplementary Table A. The average facial colors in the six conditions.**

| **Condition** | **Hue angle modification (deg)** | **∆E from the baseline** | **[a*,b*]** |
| --- | --- | --- | --- |
| Baseline | — | — | [11.70,14.85] |
| Red | 0 | 12 | [23.70,14.85] |
| Red+ | 0 | 20 | [31.70,14.85] |
| Yellow | 90 | 12 | [11.70,26.85] |
| Green | 180 | 12 | [-0.30,14.85] |
| Blue | 270 | 12 | [11.70,2.85] |

**Supplementary Table B. Hue angles and a*b*-coordinates of the background color conditions.** L=48.

| **Condition** | **Hue angle (deg)** | **Saturation** | **[a*,b*]** |
| --- | --- | --- | --- |
| Achromatic (baseline) | — | 0 | [0,0] |
| Red | 24 | 60 | [54.81,24.40] |
| Red+ | 24 | 80 | [73.08,32.54] |
| Green | 162 | 60 | [-57.06,18.54] |
| Blue | 260 | 60 | [-10.42,-59.09] |
